# Microporous Carbon and Carbon/Metal Composite Materials Derived from Bio-Benzoxazine-Linked Precursor for CO_2_ Capture and Energy Storage Applications

**DOI:** 10.3390/ijms23010347

**Published:** 2021-12-29

**Authors:** Mohamed Gamal Mohamed, Maha Mohamed Samy, Tharwat Hassan Mansoure, Chia-Jung Li, Wen-Cheng Li, Jung-Hui Chen, Kan Zhang, Shiao-Wei Kuo

**Affiliations:** 1 Department of Materials and Optoelectronic Science, Center of Crystal Research and Center for Functional Polymers and Supramolecular Materials, National Sun Yat-Sen University, Kaohsiung 80424, Taiwan; d083100006@nsysu.edu.tw (M.M.S.); lcj@g-mail.nsysu.edu.tw (C.-J.L.); 2 Chemistry Department, Faculty of Science, Assiut University, Assiut 71516, Egypt; tharout.mansour@science.au.edu.eg; 3 Department of Chemistry, National Kaohsiung Normal University, Kaohsiung 802, Taiwan; 610832003@nknu.edu.tw (W.-C.L.); t1446@nknu.edu.tw (J.-H.C.); 4 Research School of Polymeric Materials, School of Materials Science and Engineering, Jiangsu University, Zhenjiang 212013, China; zhangkan@ujs.edu.cn; 5 Department of Medicinal and Applied Chemistry, Kaohsiung Medical University, Kaohsiung 807, Taiwan

**Keywords:** polybenzoxazine, ring-opening polymerization, porous organic polymers, zeolitic imidazolate frameworks, energy storage

## Abstract

There is currently a pursuit of synthetic approaches for designing porous carbon materials with selective CO_2_ capture and/or excellent energy storage performance that significantly impacts the environment and the sustainable development of circular economy. In this study we prepared a new bio-based benzoxazine (AP-BZ) in high yield through Mannich condensation of apigenin, a naturally occurring phenol, with 4-bromoaniline and paraformaldehyde. We then prepared a PA-BZ porous organic polymer (POP) through Sonogashira coupling of AP-BZ with 1,3,6,8-tetraethynylpyrene (P-T) in the presence of Pd(PPh_3_)_4_. In situ Fourier transform infrared spectroscopy and differential scanning calorimetry revealed details of the thermal polymerization of the oxazine rings in the AP-BZ monomer and in the PA-BZ POP. Next, we prepared a microporous carbon/metal composite (PCMC) in three steps: Sonogashira coupling of AP-BZ with P-T in the presence of a zeolitic imidazolate framework (ZIF-67) as a directing hard template, affording a PA-BZ POP/ZIF-67 composite; etching in acetic acid; and pyrolysis of the resulting PA-BZ POP/metal composite at 500 °C. Powder X-ray diffraction, thermogravimetric analysis, scanning electron microscopy, transmission electron microscopy, and Brunauer–Emmett–Teller (BET) measurements revealed the properties of the as-prepared PCMC. The PCMC material exhibited outstanding thermal stability (T_d10_ = 660 °C and char yield = 75 wt%), a high BET surface area (1110 m^2^ g^–1^), high CO_2_ adsorption (5.40 mmol g^–1^ at 273 K), excellent capacitance (735 F g^–1^), and a capacitance retention of up to 95% after 2000 galvanostatic charge–discharge (GCD) cycles; these characteristics were excellent when compared with those of the corresponding microporous carbon (MPC) prepared through pyrolysis of the PA-BZ POP precursors with a ZIF-67 template at 500 °C.

## 1. Introduction

Benzoxazine (BZ) is a six-membered heterocyclic molecule, containing oxygen and nitrogen atoms, that is stable at room temperature or in humid environments; BZ derivatives are readily prepared through Mannich condensations of phenol derivatives with paraformaldehyde and primary amines in the melt or in solution [1,2,3,4,5]. Ring-opening polymerization (ROP) of the oxazine ring in a BZ precursor can be performed by applied heat, without adding any catalyst or initiator, to afford a polybenzoxazine (PBZ) as a thermosetting polymeric material [1,2,3,4,5]. PBZs are interesting materials in the industry and academia because of their excellent dimensional stability, low dielectric constants, high thermal stability, excellent adhesive strength, catalyst-free polymerization, light weight, good mechanical properties, high glass transition temperatures (*T*_g_), low surface free energies, good electrical properties, smart macromolecular design flexibility, near-zero volumetric expansion, high char yield, and rapid improvement in physical and mechanical properties at low conversion, making them useful alternatives to compared to traditional thermosetting resins (e.g., phenolic, polyimide, epoxy, bismaleimides) [6,7,8,9].

Nevertheless, the application of PBZ materials has been restricted industrially because the ROP of BZ resins to afford PBZs generally occurs at temperatures between 220 and 280 °C [10,11,12,13,14]. As a result, lowering the exothermic curing temperatures of BZ precursors has become desirable to encourage the use of these materials in industrial applications and academic settings [1,2,3,4,5]. Many reviews describe how adding a catalyst, cationic species, or initiator or incorporating various functional groups (e.g., carboxylic acids, amines, phenols) into BZ resins can accelerate the rate of polymerization or decrease the polymerization temperature [11]. Many BZs derived from renewable, naturally abundant, bio-based phenols (e.g., resveratrol, bisguaiacol-F, sesamol, diphenolic acid, magnolol, eugenol, daidzein, apigenin, guaiacol, naringenin, coumarin, vanillin, sesamol, cardanol, urushiol, and guaiacol) have been easier to prepare, have displayed superior thermal properties and higher cross-linking densities, and have undergone ROP at lower temperatures when compared with those characteristics of BZ resins derived from petroleum compounds [15,16,17,18,19,20,21,22,23,24,25,26]. For example, apigenin-, naringenin-, and daidzein-based bio-BZs have high glass transition temperatures (*T*_g_, up to 360 °C) because they allow additional crosslinking reactions to occur from the olefinic bonds in their molecular structures, leading to highly cross-linked networks after ROP of their BZ units [27,28,29]. In addition, the capability of intramolecular hydrogen bonding in these bio-based phenols leads to lower polymerization temperatures, stable latent catalysts, and improved shelf life of such BZ resins [27,29].

The development of high-energy-density electrochemical energy storage devices has been driven by the emergence and steady growth of the electric vehicle market in recent years [30,31,32]. Among the available electrochemical energy storage devices, batteries, and supercapacitors are particularly attractive for their high energy densities, high efficiencies, and low CO_2_ emissions [33,34,35,36]. Nevertheless, the sluggish nature of electron and ion transfer limits the efficiency of battery devices; furthermore, batteries suffer from overheating and dendrite formation when operated at high power, thereby presenting a great challenge in developing safe high-energy-density batteries [37,38]. Because supercapacitors can safely provide high power densities, excellent cycling stability, fast charging, and extremely long cycle life (>10^5^ cycles), they are potential replacements for batteries. Nevertheless, the energy densities of commercially available supercapacitors (ca. 5 W h kg^–1^) remain significantly lower than those of batteries (ca. 200 W h kg^–1^) [31]. Thus, extensive research efforts will be needed to develop novel materials possessing high specific surface areas that can increase the energy density of supercapacitors while maintaining their power density and cycling stability. In this regard, many porous organic polymers (POPs) featuring suitable pore size distributions—including covalent triazine frameworks (CTFs) [39], covalent organic frameworks (COFs) [40,41,42], hypercrosslinked porous polymers (HCPs) [43,44], conjugated microporous polymers (CMPs) [45,46,47,48,49,50,51,52,53], metal–organic framework [48,49,50], and ferrocene-based conjugated microporous polymers [51]—have been developed to improve the performance of supercapacitors. Supercapacitors can store energy through faradaic (pseudocapacitance) and non-faradaic [electric double-layer capacitance (EDLC)] processes [43,44,45,46,47]. The energy densities of carbon materials suitable for use in supercapacitors can be enhanced through doping with heteroatoms and/or redox moieties, thereby providing the chance to merge the features of both pseudocapacitor and EDLC mechanisms [43,44,45,46,47].

**Scheme 1:**
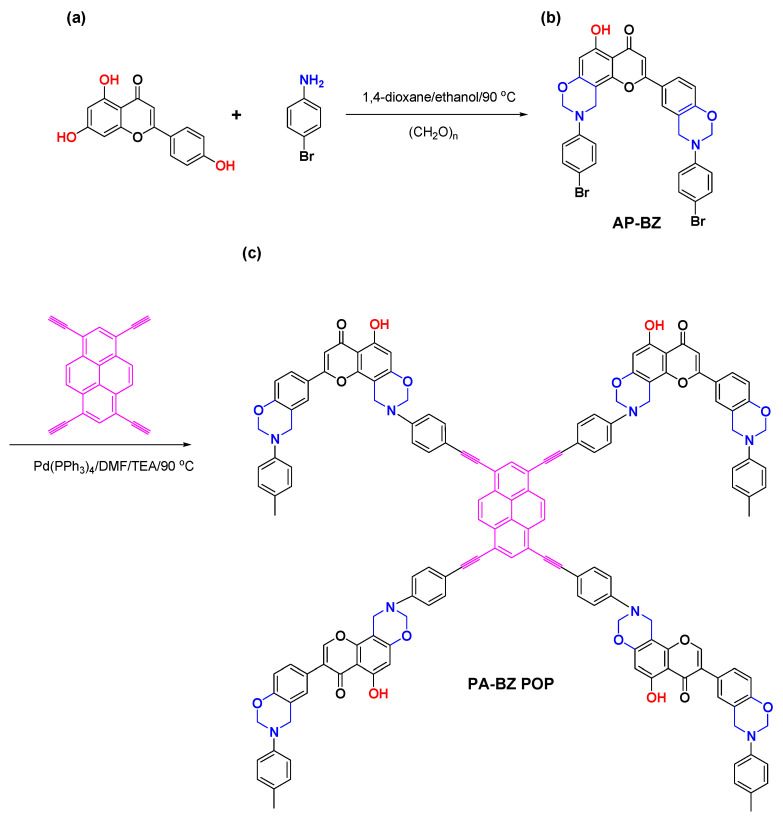
Synthesis of (**b**) AP-BZ and (**c**) PA-BZ POP precursors from (**a**) apigenin.

Taking advantage of the unique properties of BZs, POPs, and metal–organic frameworks, in this study we prepared porous carbons—a microporous carbon (MPC) and a microporous carbon/metal composite (PCMC)—and examined their properties and potential applications. We synthesized a new bio-based BZ (AP-BZ) through Mannich condensation of apigenin and then prepared a PA-BZ POP through Sonogashira coupling of AP-BZ with 1,3,6,8-tetraethynylpyrene (P-T) (Scheme 1). We used differential scanning calorimetry (DSC) and Fourier transform infrared (FTIR) spectroscopy to investigate the thermal stability and ROP of both AP-BZ and PA-BZ POP. Next, we used a zeolitic imidazolate framework (ZIF-67) as a directing template to prepare a PA-BZ POP/ZIF-67 composite; after removing the core of ZIF-67 through etching in acetic acid and pyrolyzing at 500 °C, we obtained a PCMC exhibiting outstanding thermal stability, a high Brunauer–Emmett–Teller (BET) surface area, high CO_2_ adsorption, and excellent supercapacitance, when compared with those of the corresponding MPC prepared through pyrolysis of the PA-BZ POP precursors at 500 °C.

## 2. Results and Discussion

### 2.1. Synthesis and Characterization of AP-BZ Monomer

**Figure 1:**
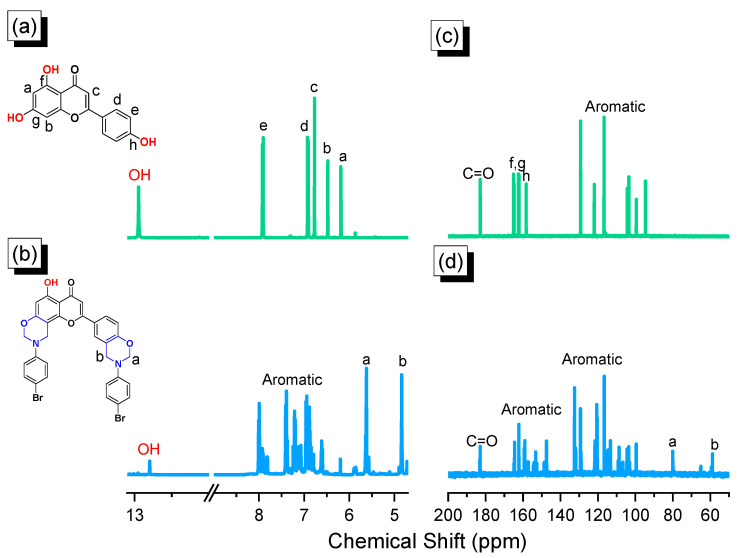
(**a**,**b**) ^1^H and (**c**,**d**) 1^3^C NMR spectra of (**a**,**c**) apigenin and (**b**,**d**) PA-BZ in DMSO-d_6_.

Scheme 1 presents our synthetic approach toward the new bio-based BZ (AP-BZ) (Scheme 1b) based on apigenin, containing a free phenolic OH group, through the one-pot reaction of apigenin (as the phenol) with 4-bromoaniline and paraformaldehyde in 1,4-dioxane and absolute EtOH at 90 °C for 24 h. When we attempted to prepare AP-BZ using DMSO as the solvent, the resulting product contained a lot of impurities and was very difficult to purify. Therefore, we performed the reaction in 1,4-dioxane and absolute EtOH as co-solvents to improve the solubility of apigenin. FTIR and NMR spectra confirmed the successful synthesis of AP-BZ. The FTIR spectrum of AP-BZ (Figure S1) featured absorption bands centered at 3380, 3025, 1668, 1581, 1500, 1240, and 940 cm^–1^, which we assign to the free phenolic OH group, the aromatic C–H unit, the C=O group, the C=C unit of the benzopyrone structure, the C=C unit in the apigenin structure, C–O–C antisymmetric stretching of the oxazine structure, and a BZ-related band, respectively. Figure 1 present the ^1^H and ^13^C NMR spectra of apigenin and AP-BZ in DMSO-d_6_, recorded at 25 °C. The ^1^H NMR spectrum of apigenin (Figure 1a) featured signals at 12.94 and 7.88–6.20 ppm, representing the phenolic OH group and aromatic protons, respectively; the ^1^H NMR spectrum of AP-BZ (Figure 1b) featured signals at 12.94, 7.97–6.63, 5.61, and 4.82 ppm, assigned to the protons of the free OH group, the aromatic ring, and the OCH_2_N and ArCH_2_O units, respectively. The ^13^C NMR spectrum of apigenin (Figure 1c) featured peaks at 182.62, 161.99, and 103.43 ppm, representing the carbon nuclei of the C=O group and benzopyrone unit. The presence of an oxazine ring in AP-BZ was confirmed through its ^13^C NMR spectrum (Figure 1d), which featured signals for carbon nuclei at 183.13, 80.08, and 44.79 ppm representing the C=O group and OCH_2_N and ArCH_2_O units, respectively. The FTIR and NMR spectral data confirmed the synthesis of AP-BZ (with a yield of 85%) and that its structure contained apigenin and oxazine units.

**Figure 2:**
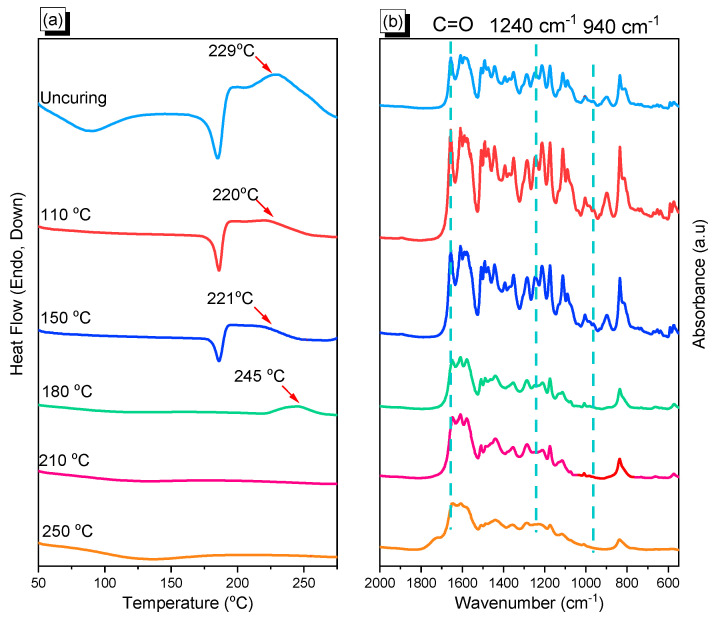
(**a**) DSC and (**b**) in situ FTIR spectral analyses of the uncured PA-BZ and the PA-BZ thermally polymerized at various curing temperatures to form poly(PA-BZ).

Next, were used DSC and in situ FTIR spectroscopy (Figure 2) to investigate the thermal ROP of the oxazine ring in the AP-BZ monomer. The DSC profile of the uncured AP-BZ (Figure 2a) revealed two types of thermal events: a sharp endothermic peak at 185 °C, due to the melting of AP-BZ, indicating its high purity, and a thermal event at 229 °C that we attribute to complete ROP of the oxazine ring in the AP-BZ structure. As reported previously, BZ monomers containing free phenolic OH groups can act as efficient catalysts that lower the exothermic curing temperature of the oxazine ring, relative to that of traditional BZ resins [54,55]. Notably, the polymerization temperature of AP-BZ was very low when compared with those of the traditional BZ monomers TPE-BZ and TPEP-BZ [52], due the acidic character of the phenolic OH group in AP-BZ accelerating the polymerization of the oxazine ring, consistent with previous studies [26]. Interestingly, after thermal curing polymerization of AP-BZ at 110 and 150 °C, the DSC profiles featured the endothermic peak of the AP-BZ monomer at 186 °C, indicating the stability of the crystalline properties of AP-BZ at these two temperatures. The maximum exothermic peak of AP-BZ monomer cured at these two temperatures had shifted to 220 and 221 °C, respectively. When we increased the thermal treatment temperature to 180 °C, the intensity of the exothermic curing peak of AP-BZ decreased, then disappearing completely when cured at 210 and 250 °C, indicating the complete curing polymerization of the oxazine ring in AP-BZ to form poly(AP-BZ) (Scheme S1). We used in situ FTIR spectroscopy to examine the thermal polymerization behavior of AP-BZ at the various temperatures (Figure 2b). As mentioned above (Figure S1 and Figure 2b), the spectrum of the uncured AP-BZ featured characteristic absorption bands for the C=O group, the C=C stretching vibration of the benzopyrone structure, C–O–C antisymmetric stretching of the oxazine ring, and a BZ-related vibration at 1581, 1500, 1240, and 940 cm^–1^, respectively. The bands at 1240 and 940 cm^–1^ representing the C–O–C antisymmetric stretching of the oxazine ring and the BZ-related vibration, respectively, remained in the FTIR spectra recorded after thermal treatment at 110 and 150 °C, but had decreased in intensity and ultimately disappeared completely when treated at 180, 210, and 250 °C, indicating that ROP of the oxazine ring in AP-BZ had occurred at these latter temperatures to form poly(AP-BZ). The DSC data were consistent with those from the FTIR spectral analyses.

**Figure 3:**
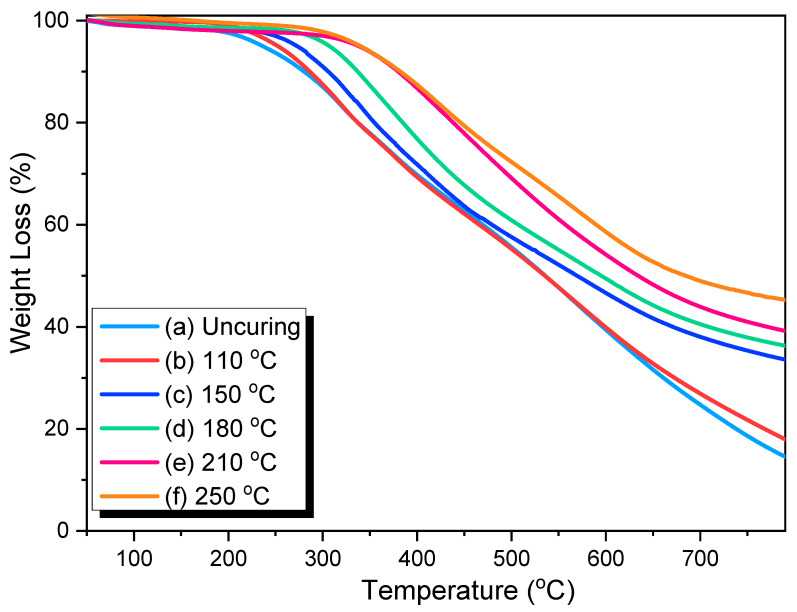
TGA analyses of the uncured PA-BZ and the PA-BZ cured at various temperatures.

We applied both the Kissinger and Ozawa equations to evaluate the activation energy (E_a_) of the AP-BZ monomer from the DSC data measured at the various heating rates (Figure S2a–c). Based on the Kissinger and Ozawa methods [56,57], we calculated values of E_a_ for the AP-BZ monomer of 23.93 and 8.36 kJ mol^–1^, respectively, suggesting low-energy activation of the polymerization of AP-BZ. Notably, our new AP-BZ displayed values of E_a_ much lower than those of other BZ monomers, bis-BZ monomers, and a previously prepared apigenin-based BZ (API-fa) [25,26]. This unique characteristic of AP-BZ during polymerization inspired us to prepare POP derivatives for application to energy storage and gas capture. To examine the thermal stability of the uncured AP-BZ and of AP-BZ after thermal treatment (at 110, 150, 180, 210, and 250 °C for 2 h each temperature), we performed TGA analyses under a N_2_ atmosphere at a heating rate of 20 °C min^–1^ (Figure 3). We used the values of T_d5_ and T_d10_ and the char yield at 800 °C to characterize the thermal stability of the uncured and the cured AP-BZ samples. Figure 3 reveals that the values of T_d5_ and T_d10_ and the char yield of the uncured AP-BZ were 237 and 280 °C and 14 wt%, respectively. As expected, both the thermal degradation temperatures and the char yield of AP-BZ increased significantly upon increasing the thermal curing temperature from 110 to 250 °C, due to the formation of crosslinked structures with increasing crosslinking density after ROP of the oxazine ring. The poly(AP-BZ) obtained after thermal curing at 250 °C had higher thermal stability—characterized by higher values of T_d5_ (340 °C) and T_d10_ (382 °C) and a higher char yield (45 wt %)—than the previously reported BZ monomers poly(AP-f), poly(BA-a), and poly(NAR-fa) [25,26].

### 2.2. Synthesis and Characterization of PA-BZ POP

**Figure 4:**
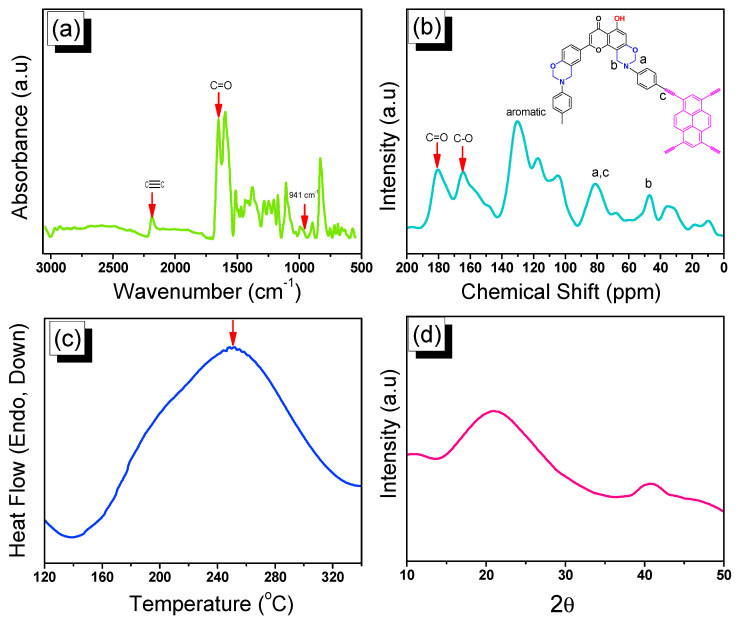
(**a**) FTIR spectrum, (**b**) solid state ^13^C CP/MAS NMR spectrum, (**c**) DSC trace, and (**d**) XRD profile of PA-BZ POP, recorded at room temperature.

Scheme 1c present the preparation of the PA-BZ POP through Sonogashira coupling of AP-BZ with P-T in the presence of a Pd catalyst in DMF/Et_3_N at 90 °C for 72 h. FTIR and ^13^C NMR solid state spectroscopy confirmed its successful synthesis. The FTIR spectrum of AP-BZ (Figure 4a), recorded at 25 °C, featured absorption bands at 3423 cm^–1^ for the phenolic OH group, 3054 cm^–1^ for the aromatic C–H units, 2192 cm^–1^ for internal and terminal -C≡C- stretching, 1645 cm^–1^ for the C=O groups, and 941 cm^–1^ for the BZ-related vibration. The solid state ^13^C CP/MAS NMR spectral profile of AP-BZ POP (Figure 4b) displayed six major peaks for the carbon nuclei in AP-BZ: at 180.27 ppm for the C=O group, 164.40 ppm for the C=C unit in the benzopyrone structure of apigenin, 128.93–103.92 ppm for the aromatic units, and 80.98–67.14 ppm for the alkyne units. Signals for the carbon nuclei of AP-BZ POP appeared at 80.98 ppm for the OCH_2_N unit and at 46.79 ppm for the ArCH_2_N unit, confirming the presence of oxazine rings. Furthermore, the maximum exothermic curing peak of the oxazine ring of the AP-BZ POP appeared at 257 °C in the DSC thermograms Figure 4c). The semicrystalline character of the PA-BZ POP was evidenced by three major diffraction peaks appearing at 10.18, 20.79, and 40.79° in the X-ray diffraction (XRD) pattern as presented in Figure 4d [12,45,58]. We also investigated the ROP of PA-BZ POP through in situ FTIR spectroscopic analyses at temperatures from 25 to 250 °C (Figure S3). The absorption bands at 1232 and 921 cm^–1^ for the BZ ring disappeared completely after thermal curing polymerization at temperatures of 210 and 250 °C, consistent with the formation of poly(PA-BZ POP) and complete opening of the oxazine ring at these curing temperatures. Scheme S2 presents a possible structure of poly(PA-BZ POP) after ROP. SEM and TEM images (Figure S4a–d) revealed irregular spherical porous and microporous structures for the surface morphologies of the PA-BZ POP. The BET surface area of the PA-BZ POP was low (25 m^2^ g^–1^), as calculated from N_2_ adsorption/desorption measurements.

### 2.3. Formation of PCMC

**Scheme 2:**
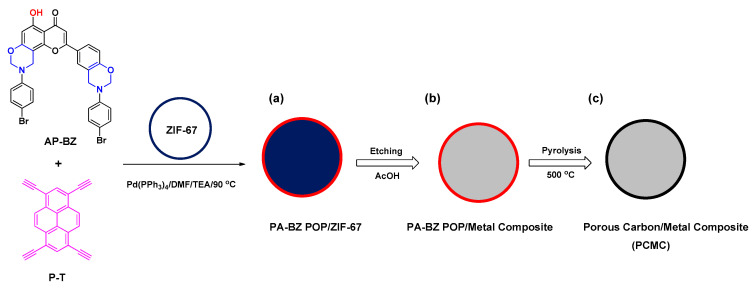
Synthesis of (**a**) PA-BZ POP/ZIF-67, (**b**) PA-BZ POP/Metal Composite and (**c**) PCMC through Sonogashira coupling reaction, etching and pyrolysis process.

Scheme 2 outlines the preparation of the PCMC. In the first step, in situ polymerization of AP-BZ and P-T through Sonogashira coupling in the presence of ZIF-67 as a director template, Pd(PPh_3_)_4_ as the catalysts, and a mixture of DMF/Et_3_N as the solvent afforded PA-BZ POP/ZIF-67 composites (Scheme 2a). Next, the PA-BZ POP/ZIF-67 composites were immersed and stirred in acetic acid for 24 h at room temperature to remove the core ZIF-76 and provide a PA-BZ POP/metal composite (Scheme 2b). Finally, the as-prepared PA-BZ POP/metal composite was carbonized by heating at a rate of 5 °C min^–1^ up to 500 °C and then maintaining that temperature for 8 h, giving the PCMC as a black solid (Scheme 2c).

**Figure 5:**
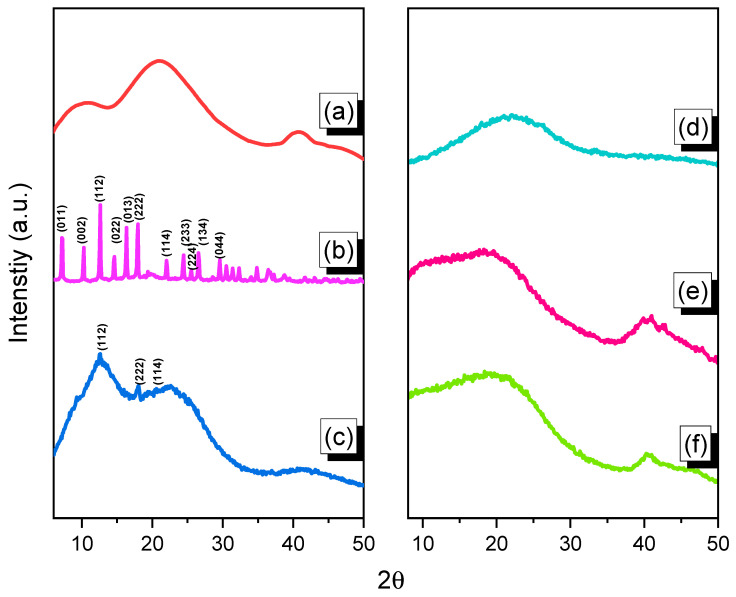
XRD profiles of (**a**) PA-BZ POP, (**b**) ZIF-67, (**c**) PA-BZ POP/ZIF-67, (**d**) PA-BZ POP/metal composite, (**e**) MPC, and (**f**) PCMC, recorded at room temperature.

**Figure 6:**
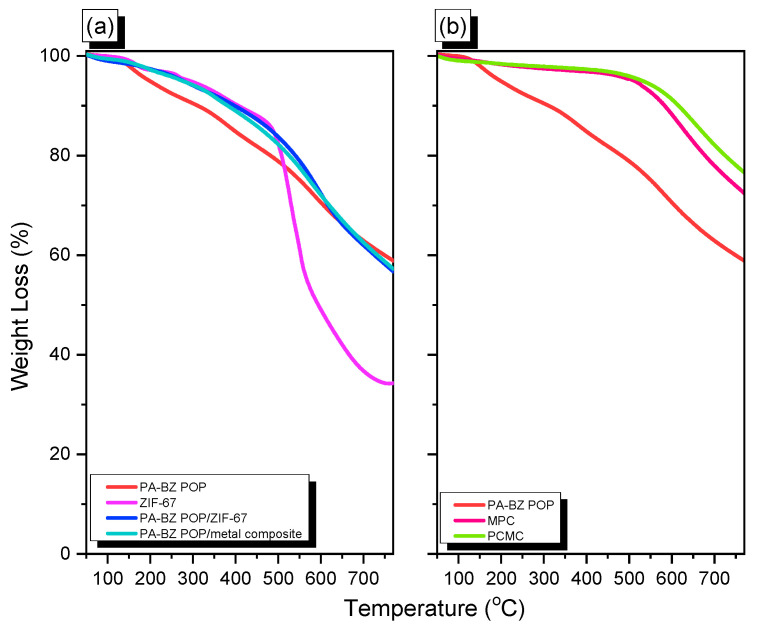
TGA profiles of the (**a**) PA-BZ POP, ZIF-67, PA-BZ POP/ZIF-67, and PA-BZ POP/metal composite and (**b**) PA-BZ POP, MPC, and PCMC.

This new PCMC material was characterized through XRD, TGA, and BET measurements. Figure 5 presents the XRD patterns of PA-BZ POP, ZIF-67, PA-BZ POP/ZIF-67, PA-BZ POP/metal composite, MPC, and PCMC, each recorded at room temperature. As mentioned earlier, the PA-BZ POP had semicrystalline character (Figure 4d and Figure 5a). The XRD pattern of ZIF-67 featured many sharp peaks at values of 2θ of 7.23, 10.17, 12.58, 14.64, 16.27, 17.97, 22.04, 24.35, 25.47, 26.59, and 28.63°, representing the (011), (002), (112), (022), (013), (222), (114), (233), (224), (134), and (044) planes, respectively, suggesting its successful preparation with a highly crystalline and long-range-ordered structure (Figure 5b) [59,60]. The pattern of the PA-BZ POP/ZIF-67 composite featured three major diffraction peaks at 12.58, 17.97, and 22.04° corresponding to the (112), (222), and (114) planes, respectively (Figure 5c), confirming the formation of a core/shell structure (Scheme 2a). Using acetic acid to remove the ZIF-67 structure from the PA-BZ POP/ZIF-67 composite produced the amorphous PA-BZ POP/metal composite (Figure 5d). Both the MPC and PCMC materials possessed graphitic carbon structures with diffraction peaks appearing in 20.21° and in the range 39.91–42.96° attributed to the presence of layered carbon structures in these two samples (Figure 5e,f) [61,62,63,64,65]. Finally, we examined the thermal stabilities of these six samples through TGA under a N_2_ atmosphere (Figure 6). The values of T_d5_ and T_d10_ and the char yield of PA-BZ-POP were 198 °C, 311 °C, and 57.7 wt%, respectively; for ZIF-67, they were 293 °C, 407 °C, and 35.1 wt%, respectively; for the PA-BZ POP/ZIF-67 composite they were 278 °C, 398 °C, and 55.2 wt%, respectively; for the PA-BZ POP/metal composite they were 275 °C, 382 °C, and 55.7 wt% respectively; for the MPC they were 513 °C, 583 °C, and 70.9 wt%, respectively; and for the PCMC they were 532 °C, 660 °C, and 75.0 wt%, respectively. The TGA data presented two interesting phenomena: First, the thermal stabilities of PA-BZ POP/ZIF-67 and PA-BZ POP/metal composite were outstanding when compared with that of the pure PA-BZ POP, due to the presence of ZIF-67 and the formation of a composite after removing the ZIF-67 framework. Second, the thermal degradation temperature and char yield of the PCMC were higher than those of the MPC, presumably because the former had a nanoparticle-embedded porous carbon structure that improved the thermal stability of this material.

**Figure 7:**
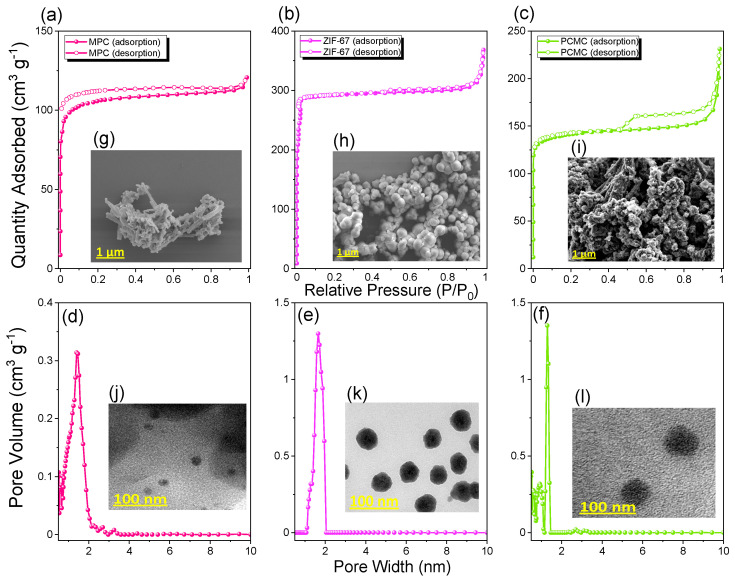
(**a**–**c**) N_2_ adsorption/desorption isotherms of (**a**) MPC, (**b**) ZIF-67, and (**c**) PCMC. Pore size distribution of (**d**) MPC, (**e**) ZIF-76, and (**f**) PCMC. (**g**–**i**) SEM and (**j**–**l**) TEM images of (**g**,**j**) MCP, (**h**,**k**) ZIF-67, and (**i**,**l**) PCMC.

The porosities and pore size diameter of MPC, ZIF-67, and PCMC materials were identified using N_2_ sorption isotherms (Figure 7). The pure MPC material provided a type I isotherm with an upsurge in the N_2_ absorption curve occurring at a value of P/P_0_ of less than 0.10, and only a slight upsurge in the range 0.1–1 (Figure 7a). The ZIF-67 template (Figure 7b) also exhibited a type I isotherm, with very large upsurge in the N_2_ absorption curve at a value of P/P_0_ of less than 0.10, a slight upsurge in the range 0.03–0.85, and finally another upsurge in the range 0.85–1. In contrast, the PCMC (Figure 7c) provided a type IV isotherm, with an upsurge in the N_2_ absorption curve at a value of P/P_0_ of less than 0.10, a slight upsurge in the range 0.03–0.49, and finally a narrow hysteresis loop in the range 0.49–1, suggesting the presence of micropores and mesopores inside this material. The BET surface areas were 403 m^2^ g^–1^ for MPC and 930 m^2^ g^–1^ for ZIF-67; the PCMC possessed the highest BET surface area (1110 m^2^ g^–1^), presumably because of the growth of ZIF-67 particles inside the pores of the PA-BZ POP material, and after removing the ZIF-67 via etching process and after further activation at 500 °C dramatically improved the porosity and surface area [51]. The pore size distribution (PSD) curves of MPC, ZIF-67, and PCMC (Figure 7d–f) revealed average pore size of 1.34, 1.65, and 1.29 nm, respectively, confirming their microporous structures. Furthermore, SEM and TEM images revealed that the surfaces of MPC, ZIF-67, and PCMC structures featured the aggregation of irregular nanorods and spheres, regular uniform spheres, and large spheres, respectively, confirming their microporosity (Figure 7g–l). In addition, SEM combined with energy-dispersive spectroscopy (EDS) mapping analysis confirmed the presence of C, N, O, and Co in the PCMC material, as shown in Figure S5.

**Figure 8:**
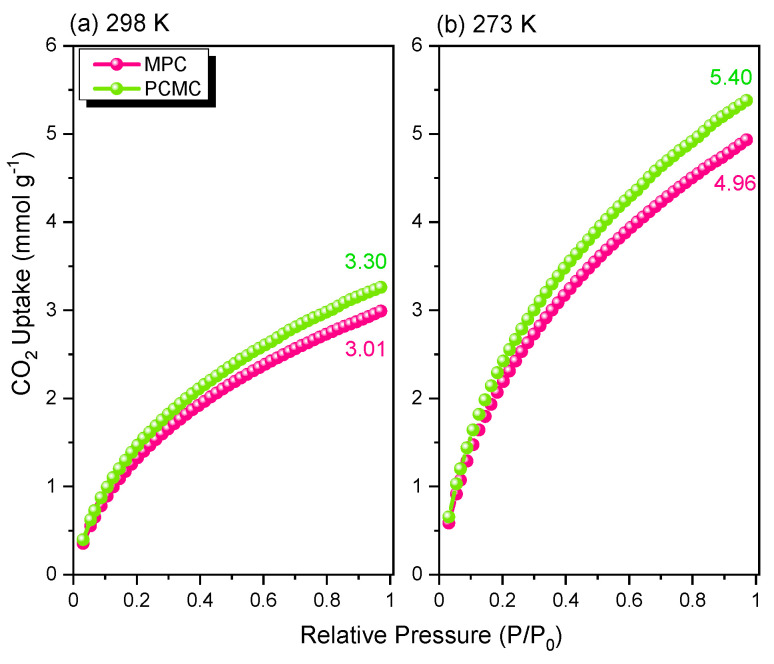
CO_2_ adsorption profiles of MPC and PCMC, recorded at (**a**) 298 and (**b**) 273 K.

Because of the high BET surface areas and thermal stabilities of the MPC and PCMC, we expected that these materials could be applied for CO_2_ adsorption and energy storage. We performed CO_2_ uptake measurements for the MPC and PCMC at 273 and 298 K, respectively (Figure 8). The amount of CO_2_ captured by the MPC and PCMC were 3.01 and 3.30 mmol g^–1^, respectively, at 298 K (Figure 8a) and 4.96 and 5.40 mmol g^–1^, respectively, at 273 K (Figure 8b). We attribute the higher CO_2_ uptake for PCMC to its higher surface area (1110 m^2^ g^–1^) and abundance of micropores that provided suitable spaces for CO_2_ uptake. Furthermore, because of its high surface area and large pores, the CO_2_ adsorption of our PCMC was excellent when compared with that of other previously reported porous materials, including poly(TPE-TPE-BZ), poly(Py-TPE-BZ), BPOP-1, the BZ-linked material BoxPOP-1, the ferrocene-containing material Fc-CMP-1, A6CMP-3, CTF-HUST-3, the triphenylamine-based polymer PTPA-3, CTHP-3, and COF-102 [66,67,68,69,70,71,72,73,74].

### 2.4. Electrochemical Performance

**Figure 9:**
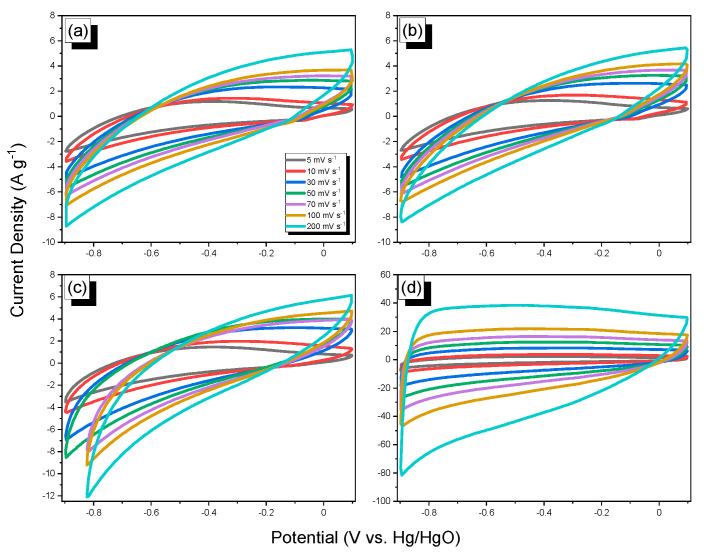
CV curves of (**a**) PA-BZ POP, (**b**) PA-BZ POP/metal composite, (**c**) MPC, and (**d**) PCMC.

We examined the electrochemical performance of our samples through cyclic voltammetry (CV) and galvanostatic charge/discharge (GCD) measurements in a three-electrode system in 1 M aqueous KOH. Figure 9a–d display the CV curves of our samples, recorded at various sweep rates from 5 to 200 mV s^–1^ in the potential window from 0.10 to 0.90 V (vs. Hg/HgO). The CV curves of all samples were rectangular in shape, even when recorded at the highest scan rate of 200 mV s^–1^, implying that the capacitive response originated mainly from EDLC, with minor pseudocapacitance. The pseudocapacitance arose from the presence of various types of oxygen and nitrogen species. The integrated area of the PA-BZ POP sample was higher than those of the other samples, revealing its superior electrochemical performance. Furthermore, the current density increased upon increasing the sweep rate, while retaining the shapes of these CV curves, suggestion facile kinetics and good rate capability [45,46,75].

**Figure 10:**
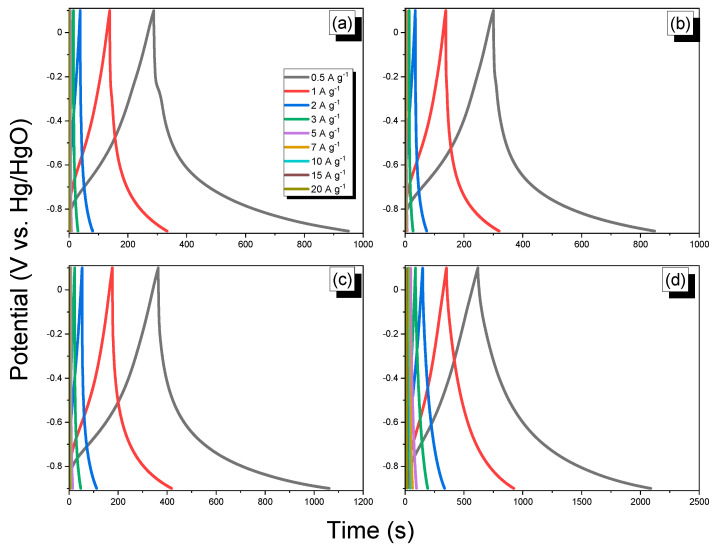
GCD performance of (**a**) P-AP-BZ POP, (**b**) PA-BZ POP/metal composite, (**c**) MPC, and (**d**) PCMC.

Figure 10a–d present the GCD curves of our samples, determined at various current densities. The GCD curves of all of the samples were triangular in shape, with only a slight bend, indicating both EDLC and pseudocapacitive characteristics [45,46]. After calcination at 500 °C, the discharging times of both the MPC and PCMC samples were longer than those of the PA-BZ POP and PA-BZ POP/metal composite (Figure 10a–d), indicating that calcination had an enhancement effect on the capacitance of our MPC and PCMC. 

**Figure 11:**
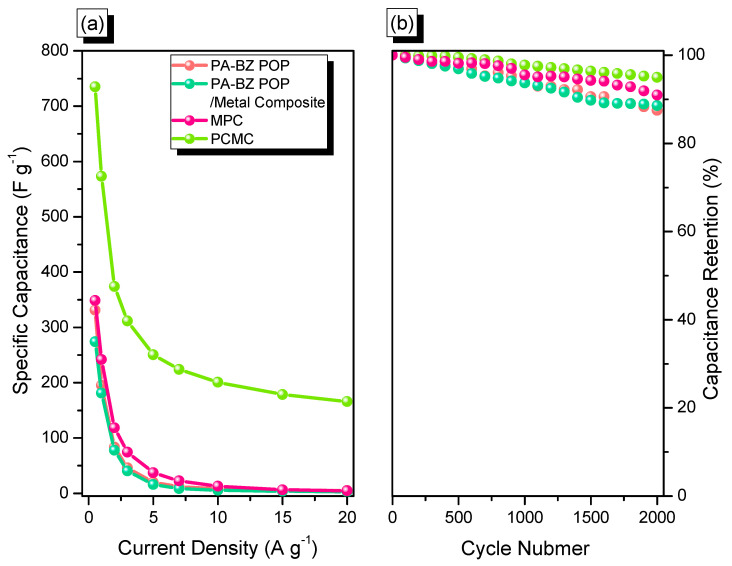
(**a**) Specific capacitance and (**b**) stability tests of PA-BZ POP, PA-BZ POP/metal composite, MPC, and PCMC.

Figure 11a displays the specific capacitances of all of the samples, determined from GCD curves using Equation (S1). The capacitance of the PCMC was excellent (735 F g^–1^) and highest among the tested samples (PA-BZ POP: 331 F g^–1^; PA-BZ POP/metal composite: 274 F g^–1^; PCM: 349 F g^–1^) at a current density of 0.5 A g^–1^. This behavior can be explained in terms of a higher surface area (1113 m^2^ g^–1^), the existence of microporosity (average pore size: 1.5 nm), and the presence of various types of nitrogen and oxygen heteroatoms (primarily pyridinic N species; C=O and phenolic OH groups), thereby enabling ready access of electrolytes to the electrode surface [45,46]. In addition, the capacitance of the PCMC was excellent when compared with those of other porous materials (Table S1). Furthermore, upon increasing the current density from 0.5 to 20 A g^–1^, the specific capacitances of all of the CMP samples decreased, due to there being insufficient time for ionic diffusion and adsorption inside the smallest pores within the large particles at high current densities [45,46]. We tested the durability of all of our samples through GCD measurements performed over 2000 cycles at a current density of 10 A g^–1^ (Figure 11b). The PA-BZ POP, PA-BZ POP/metal composite, MCP, and PCMC samples displayed good cycling stability, with 87.5, 88.6, 91.0, and 95.0% retention, respectively, of their original capacitances after 2000 cycles, indicating excellent cycling stability in 1 M KOH as the electrolyte. Recently, we prepared ferrocene-derived conjugated microporous polymers (FFC-CMPs) and then formed an inclusion complex with a BZ-linked β-cyclodextrin (Py-FFC-CMP/CD-BZ) [12]; this material exhibited capacitance of only 46 F g^–1^ at 0.5 A g^–1^. In addition, we have previously prepared a series of CMPs through Sonogashira–Hagihara cross-couplings of a tetrabenzonaphthalene (TBN) monomer with tetraphenylethylene (TPE), pyrene (Py), and carbazole (Car) units; we then mixed them with highly conductive single-walled carbon nanotubes (SWCNTs) to improve their conductivity [45], with the TBN-Py-CMP/SWCNT composite exhibiting high capacitance (430 F g^–1^) at a current density of 0.5 A g^–1^ because of strong π-stacking of the SWCNTs with the CMPs [45]. In addition, we have previously prepared two-hybrid porous POSS-F-POIP and POSS-A-POIP materials through Heck reactions of octavinylsilsesquioxane (OVS) with brominated fluorene (F–Br_2_) and anthraquinone (A-Br_2_) derivatives, respectively [47]. These POSS-A-POIP and POSS-F-POIP materials exhibited specific capacitances of 152.5 and 36.2 F g^–1^, respectively, at 0.5 A g^–1^. The higher capacitance of POSS-A-POIP was due to the faradaic reaction of anthraquinone and the π-conjugated system [47]. Shao et al. [76] prepared N-doped Cobalt@graphitized carbon material (ZC600) derived from ZIF-67 assisted polyvinylidene fluoride hollow fiber membrane by a simple anaerobic calcination method at a relative low temperature (600 °C). the obtained carbon material (ZC600) showed a specific capacitance of 652 F g^−1^ at the current density of 1 A g^−1^ in three electrode system. After 20,000 cycles, the cycling retention rate was 97.1% at 10 A g^−1^. Lei et al. [77] prepared ZIF-67/SiO_2_/RF-M composite material through one-pot method using ZIF-67, TEOS, resorcinol and formaldehyde in alkaline solution. After carbonization and remove of silica, the N-doped porous carbon (Carbon-ZSR-M) was obtained. The obtained Carbon-ZSR showed a specific capacitance of 305 at 1 A g^−1^, due to the highly conductive and large specific surface area. We used Equations (S2) and (S3) to calculate the energy and power densities, respectively, of our materials to construct the Ragone plot (Figure S6). The energy and power densities of the PCMC were good, and higher than those of the PA-BZ POP, PA-BZ POP/metal composite, and MPC.

## 3. Experimental Section

### 3.1. Materials

Tetrakis(triphenylphosphine)palladium(0) [Pd(PPh_3_)_4_, 99%], 2-methylimidazole (99%), paraformaldehyde (95%), 4-bromoaniline (97%), apigenin (97%), and triphenylphosphine (PPh_3_, 99%) was obtained from Sigma–Aldrich (Burlington, MA, USA). Triphenylphosphine (PPh_3_), methanol (MeOH), ethanol (EtOH), tetrahydrofuran (THF), *N*,*N*-dimethylformamide (DMF), triethylamine (Et_3_N, 99.5%), sodium hydroxide (NaOH, 97%), Co(NO_3_)_2_.6H_2_O (99%), and copper iodide (CuI, 99.5%) were purchased from Alfa Aesar (Haverhill, MA, USA). 1,3,6,8-Tetrabromopyrene (Py-Br_4_) and P-T were prepared using previously reported procedures [52,53].

### 3.2. Preparation of AP-BZ

A solution of apigenin (2.00 g, 7.40 mmol), 4-bromoaniline (3.82 g, 22.2 mmol), and (CH_2_O)*_n_* (1.11 g, 37.0 mmol) in 1,4-dioxane (60 mL) and absolute EtOH (40 mL) was heated at 100 °C with gentle stirring for 24 h under the N_2_ atmosphere. After cooling the mixture to 25 °C, the resulting precipitate was filtered off and the solvents evaporated under vacuum to afford a yellow powder. Then, the yellow precipitate was washed with NaOH solution (0.1 M) and they recrystallized from 1,4-dioxane:EtOH (1:1) to give a yellow solid (3.00 g, 85%). FTIR (KBr, cm^–1^): 3380 (phenolic OH), 3025 (aromatic C–H), 1656 (C=O), 1581 (C=C of benzopyrone), 1500 (C=C stretching of apigenin), 1240 (C–O–C antisymmetric stretching), 940 (BZ-related band). ^1^H NMR (500 MHz, DMSO-*d*_6_, δ, ppm): 12.94 (s, OH), 7.97–6.63 (aromatic protons), 5.61 (s, ArCH_2_O), 4.82 (OCH_2_N). ^13^C NMR (125 MHz, DMSO-*d*_6_, δ, ppm): 183.13 (C=O), 162.50, 163.61, 158.75, 157.65, 153.88, 144.82, 132.67, 130.02, 120.22, 116.98, 113.74, 109.39, 103.94, 99.08, 80.08 (s, ArCH_2_O), and 45.00 (OCH_2_N).

### 3.3. Preparation of PA-BZ POP

A solution of AP-BZ (0.464 g, 0.70 mmol), P-T (0.10 g, 0.35 mmol), Pd(PPh_3_)_4_ (40 mg, 0.035 mmol), PPh_3_ (9.0 mg, 0.046 mmol), and CuI (7.0 mg, 0.037 mmol) in dry DMF (14 mL) and Et_3_N (14 mL) was degassed under the N_2_ atmosphere and then heated under reflux at 110 °C for 72 h. The orange precipitate was filtered off, washed thoroughly with THF, MeOH, and DMF, and then dried for 48 h in an oven. FTIR (KBr, cm^–1^): 3060 (aromatic C–H), 2192 (C≡C), 1600 (C=C).

### 3.4. Preparation of Zeolitic Imidazolate Frameworks Containing Co (ZIF-67)

Solution A was formed by dissolving 2-methylimidazole (224 mmol, 18.4 g) in absolute MeOH (15 mL). The solution B was formed from Co(NO_3_)_2_·6H_2_O (12.5 mmol, 3.64 g) in absolute MeOH (15 mL). Both solutions were stirred for 25 min at 25 °C. After 20 min, solution A was poured slowly into solution B to form a purple solution, which was kept for 24 h at 25 °C to give a purple solid. This solid was filtered off and washed with MeOH to afford ZIF-67 as a purple powder.

### 3.5. Preparation of PA-BZ POP/ZIF-67

A solution of AP-BZ (0.464 g, 0.70 mmol), P-T (0.100 g, 0.35 mmol), Pd(PPh_3_)_4_ (40 mg, 0.035 mmol), PPh_3_ (9.00 mg, 0.046 mmol), ZIF-67 (0.100 g), and CuI (7.0 mg, 0.037 mmol) in dry DMF (14 mL) and Et3N (14 mL) was degassed under the N_2_ atmosphere and then heated under reflux at 110 °C for 72 h. The orange precipitate was filtered off, washed thoroughly with THF, MeOH, and DMF, and then dried in an oven for 48 h.

### 3.6. Preparation of MPC

AP-BZ POP was heated in an ampoule under a N_2_ atmosphere at a heating rate of 1 °C min^–1^ up to 500 °C and then this temperature was maintained for 8 h. After cooling to room temperature, the black material was collected to afford the MPC.

### 3.7. Preparation of PCMC

AP-BZ POP/ZIF-67 (0.20 g) was immersed and stirred in acetic acid (30 mL) for 24 h at 25 °C to remove the ZIF-67. The obtained solid was filtered off and dried at 100 °C for 24 h. The brown material was heated at a rate of 1 °C min^–1^ up to 500 °C and then this temperature was maintained for 8 h under a N_2_ atmosphere. After cooling to room temperature, the resulting black material was collected to afford the PCMC (Scheme 2).

## 4. Conclusions

We prepared a new bio-based BZ (AP-BZ) through Mannich condensation of apigenin (as the phenol) with 4-bromoaniline and paraformaldehyde. We then used Sonogashira coupling of AP-BZ with P-T to afford a new porous polymer, PA-BZ POP. Finally, we synthesized a spherically shaped PCMC through Sonogashira coupling in the presence of a zeolitic imidazolate framework as a directing template, affording PA-BZ and after etching and pyrolysis processes. Our as-prepared PCMC material exhibited outstanding thermal stability, a high BET surface area (1110 m^2^ g^–1^), high CO_2_ adsorption (5.40 mmol g^–1^ at 273 K), excellent capacitance (735 F g^–1^), and capacitance retention of up to 95% after 2000 galvanostatic charge/discharge cycles; these characteristics were superior to those of the corresponding MPC and other porous materials.

## Data Availability

The data presented in this study are available on request from the corresponding author. The data are not publicly available due to privacy.

## Supplementary Materials

The following are available online at https://www.mdpi.com/article/10.3390/ijms23010347/s1.
