# Supplementary material for: Microporous Carbon and Carbon/Metal Composite Materials Derived from Bio-Benzoxazine-Linked Precursor for CO2 Capture and Energy Storage Applications
Source: Int J Mol Sci. 2021 Dec 29;23(1):347. doi: 10.3390/ijms23010347 (PMC8745757; doi:10.3390/ijms23010347)
Supplement: Supplementary file 1 [file ijms-23-00347-s001.zip › ijms-1507423-supplementary.pdf]

Supporting Information for

Microporous Carbon and Carbon/Metal Composite Materials Derived from Bio-Benzoxazine-Linked Precursor for CO_2 Capture and Energy Storage Applications

Mohamed Gamal Mohamed[1,2,*,†] Maha Mohamed Samy[1,2,†], Tharwat Hassan Mansoure[2], Chia-Jung Li[1], Wen-Cheng Li[3], Jung-Hui Chen[3], Kan Zhang[4], and Shiao-Wei Kuo[1,5,*]

[1] Department of Materials and Optoelectronic Science, Center of Crystal Research and Center for Functional Polymers and Supramolecular Materials, National Sun Yat-Sen University, Kaohsiung 80424, Taiwan; d083100006@nsysu.edu.tw (M.M.S); lcj@g-mail.nsysu.edu.tw (C.C.L.)

[2] Chemistry Department, Faculty of Science, Assiut University, Assiut 71516, Egypt; tharout.mansour@science.au.edu.eg (T.H.M.)

[3] Department of Chemistry, National Kaohsiung Normal University, Kaohsiung 802, Taiwan; 610832003@nknu.edu.tw (W.C.L.); t1446@nknu.edu.tw (J.H.C.)

[4] Research School of Polymeric Materials, School of Materials Science and Engineering, Jiangsu University, Zhenjiang 212013, China; zhangkan@ujs.edu.cn (K.Z.)

[5] Department of Medicinal and Applied Chemistry, Kaohsiung Medical University, Kaohsiung 807, Taiwan

*Correspondence: Correspondence: mgamal.eldin12@aun.edu.eg (M.G.M.); (kuosw@faculty.nsysu.edu.tw (S.-W.K.)

† These authors contributed equally to this work.

Characterization

FTIR spectra were collected using a Bruker Tensor 27 FTIR spectrophotometer at a resolution of 4 cm^{-1} and the KBr disk method. ^{13}C nuclear magnetic resonance (NMR) spectra were recorded using an INOVA 500 instrument, with DMSO as the solvent and tetramethylsilane (TMS) as the external standard; chemical shifts are reported in parts per million (ppm). The thermal stabilities of the samples under N_2 were measured using a TG Q-50 thermogravimetric analyzer; the cured sample (ca. 5 mg) was placed in a Pt cell and then heated at 20 °C min^{-1} from 100 to 800 °C under a N_2 flow of 60 mL min^{-1}. Wide-angle X-ray diffraction (WAXD) patterns were measured at the wiggler beamline BL17A1 of the National Synchrotron Radiation Research Center (NSRRC), Taiwan; a triangular bent Si (111) single crystal was used to obtain a monochromated beam having a wavelength (λ) of 1.33 Å. The morphologies of the polymer samples were examined through field emission scanning electron microscopy (FE-SEM; JEOL JSM7610F) and transmission electron microscopy (TEM), using a JEOL-2100 microscope operated at an accelerating voltage of 200 kV. BET surface areas and porosimetry measurements of the samples (ca. 40–100 mg) were performed using a BEL Master^{TM} instrument and BEL sim^{TM} software (v. 3.0.0); N_2 adsorption and desorption isotherms were generated through incremental exposure to ultrahigh-purity N_2 (up to ca. 1 atm) in a liquid N_2 (77 K) bath; surface parameters were calculated using the BET adsorption models in the instrument's software. The pore sizes of the prepared samples were determined using nonlocal density functional theory (NLDFT).

Electrochemical Analysis

Working Electrode Cleaning: Prior to use, the glassy carbon electrode (GCE) was polished several times with 0.05-µm alumina powder, washed with EtOH after each polishing step, cleaned through sonication (5 min) in a water bath, washed with EtOH, and then dried in air.

Electrochemical Characterization: The electrochemical experiments were performed in a three-electrode cell using an Autolab potentiostat (PGSTAT204) and 1 M KOH as the aqueous electrolyte. The GCE was used as the working electrode (diameter: 5.61 mm; 0.2475 cm^2); a Pt wire was used as the counter electrode; Hg/HgO (RE-1B, BAS) was the reference electrode. All reported potentials refer to the Hg/HgO potential. A slurry was prepared by dispersing the sample (45 wt. %), carbon black (45 wt. %), and Nafion (10 wt. %) in a mixture of (EtOH/ H_2O) (200 μL: 800 μL) and then sonicating for 1 h. A portion of this slurry (10 μL) was pipetted onto the tip of the electrode, which was then dried in air for 30 min prior to use. The electrochemical performance was studied through CV at various sweep rates (5–200 mV s^{-1}) and through the GCD method in the potential range from 0 to –1.00 V (vs. Hg/HgO) at various current densities (0.5–20 A g^{-1}) in N_2 saturated 1 M KOH as the aqueous electrolyte solution.

The specific capacitance was calculated from the GCD data using the equation:

$$C_s = (I\Delta t)/(m\Delta V) \quad \textbf{(S1)}$$

Where C_s (F g^{-1}) is the specific capacitance of the supercapacitor, I (A) is the discharge current, ΔV (V) is the potential window, Δt (s) is the discharge time, and m (g) is the mass of the NPC on the electrode. The energy density (E, W h kg^{-1}) and power density (P, W kg^{-1}) were calculated using the equations.

$$E = 1000C(\Delta V)^2/(2 \times 3600) \quad \textbf{(S2)}$$

$$P = E/(t/3600) \quad \textbf{(S3)}$$

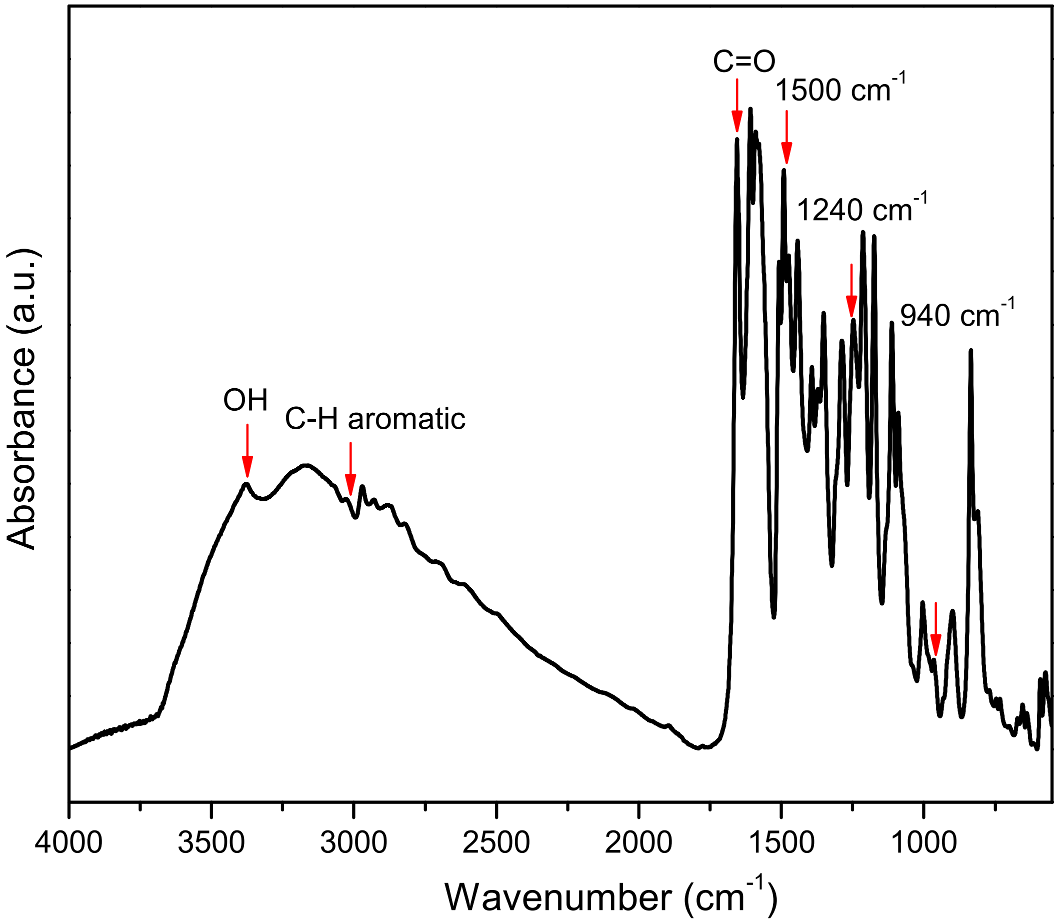

Figure S1: FTIR profile of AP-BZ.

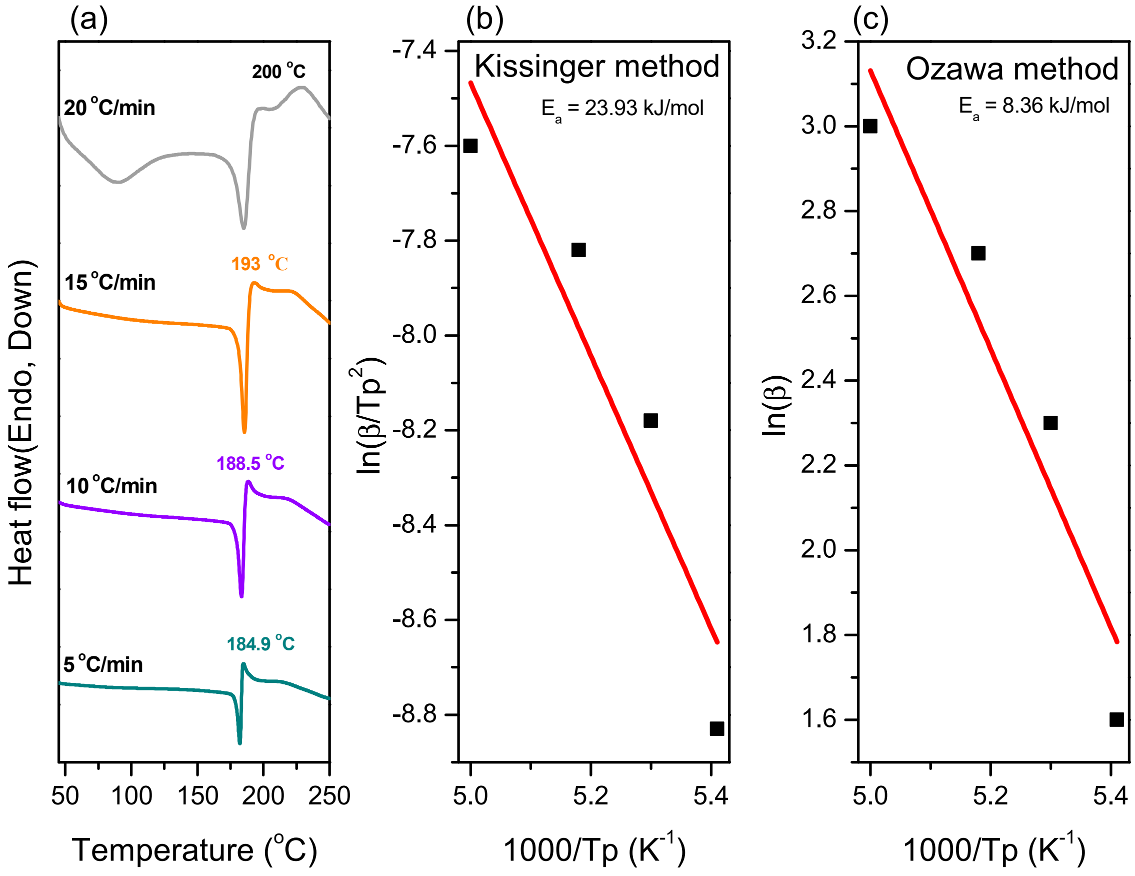

Figure S2: (a) DSC analysis of PA-BZ at different heating rates, (b and c) activation energy of PA-BZ by using Kissinger and Ozawa methods.

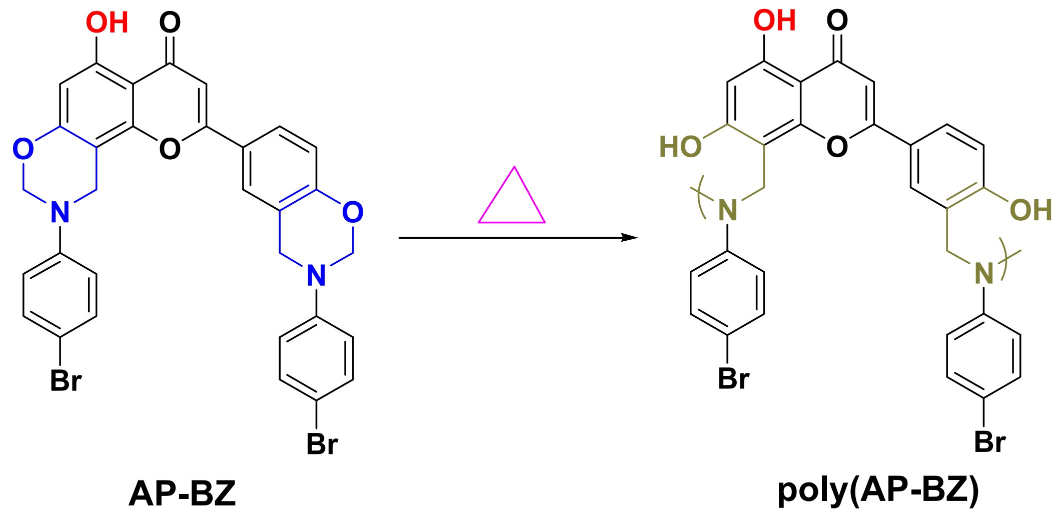

Scheme S1: Preparation of poly(AP-BZ) from AP-BZ after thermal curing polymerization.

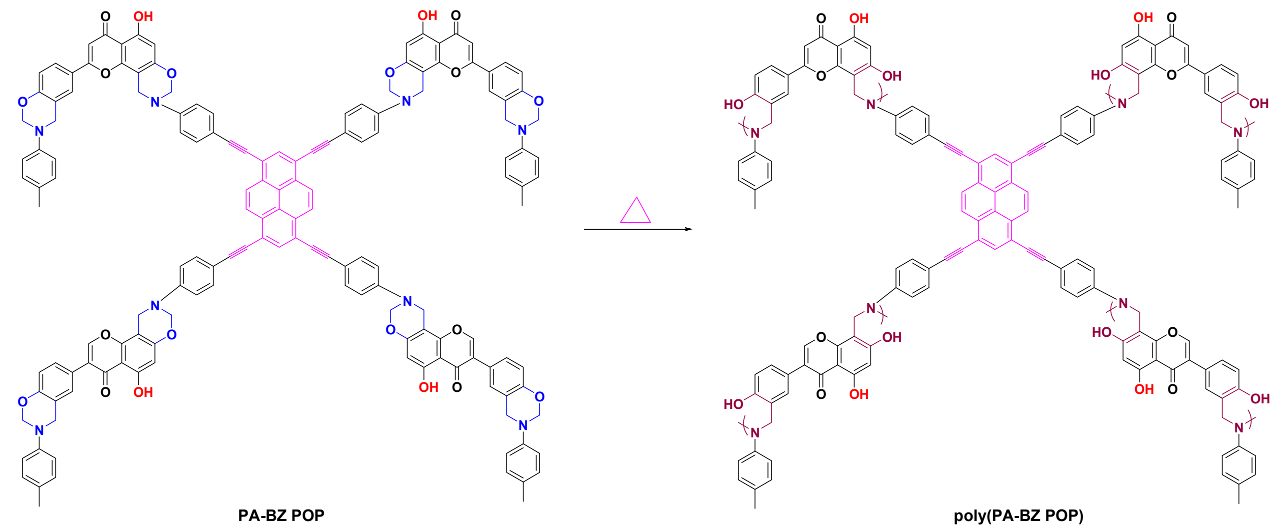

Scheme S2: Preparation of poly(PA-BZ POP) from PA-BZ POP after thermal curing polymerization.

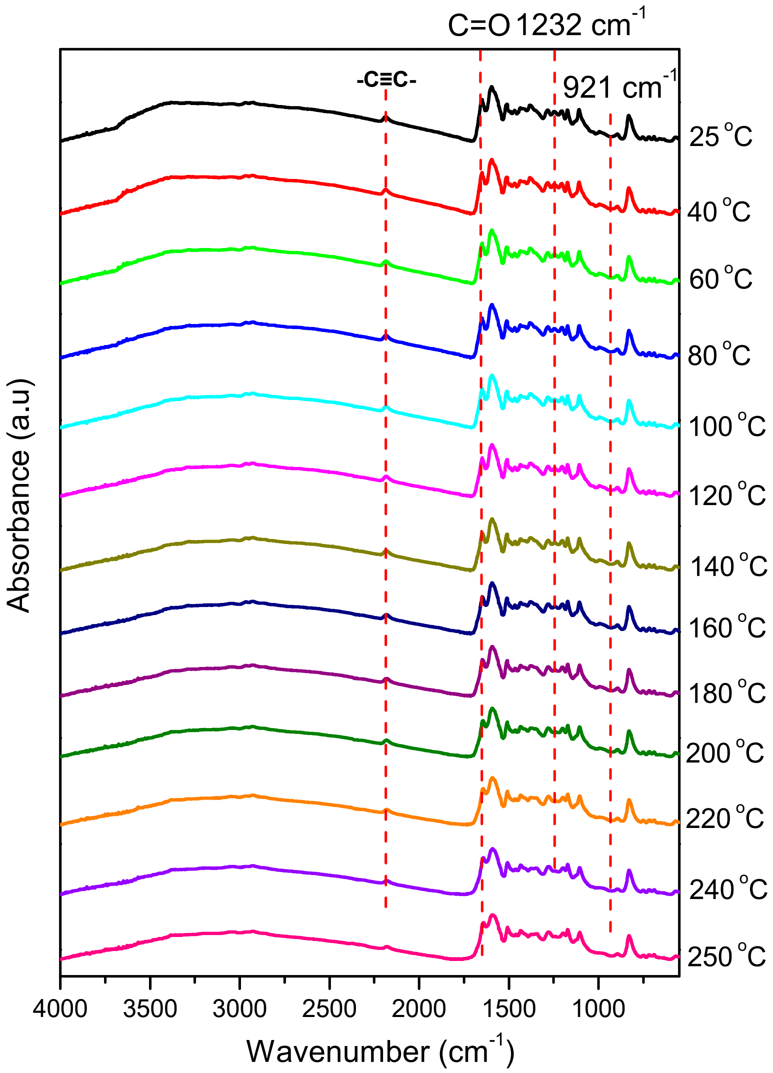

Figure S3: In situ FTIR polymerization of PA-BZ POP.

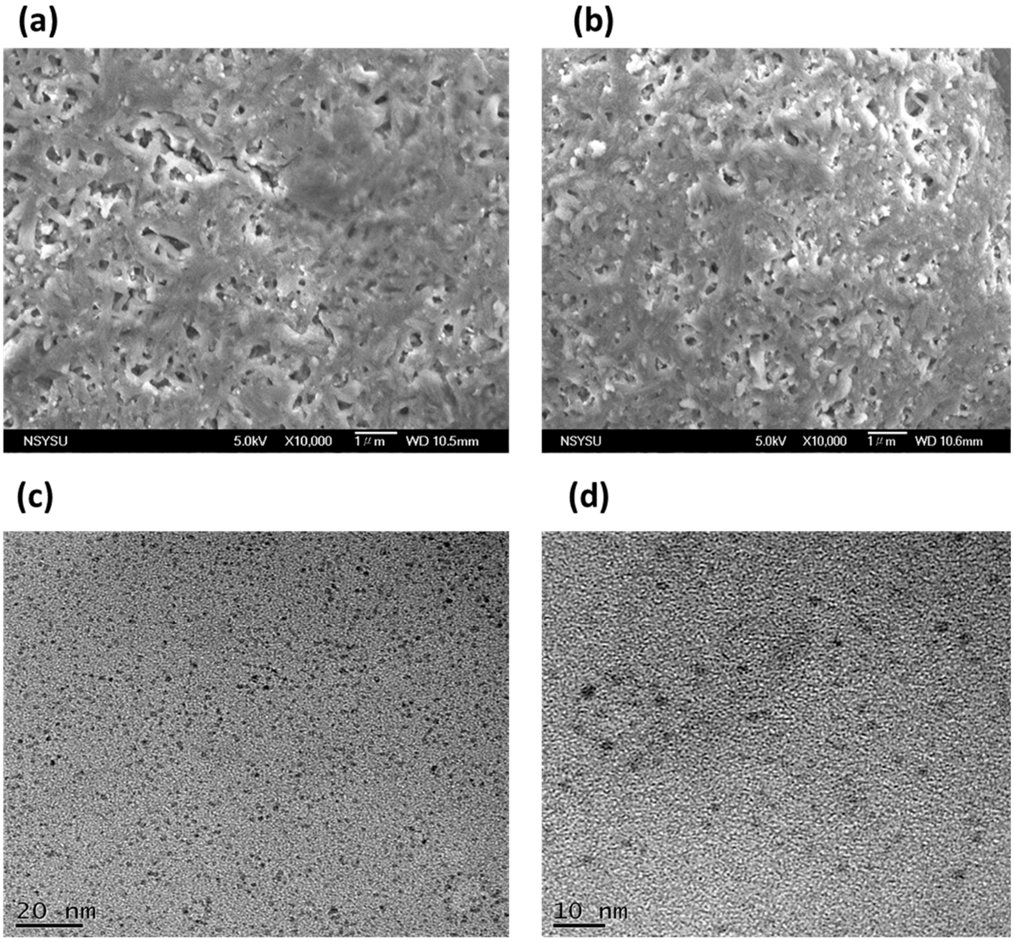

Figure S4: SEM (a, b) and TEM (c, d) images of PA-BZ POP.

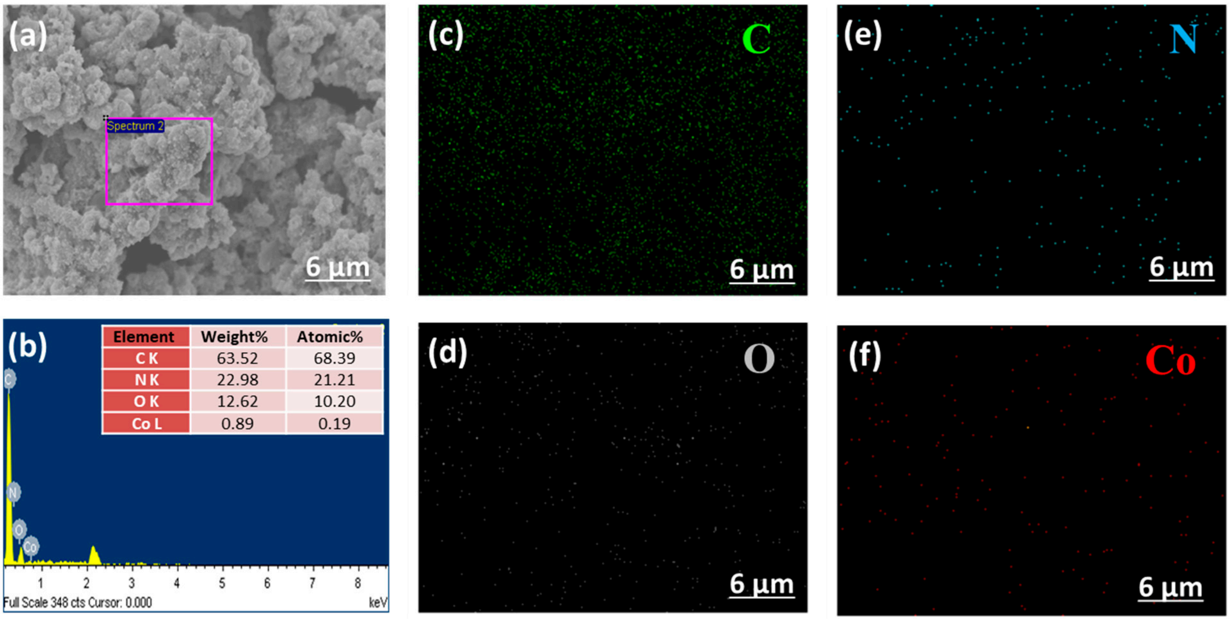

Figure S5. SEM-EDS mapping images of C, N, O and Co for the PCMC sample (a-f).

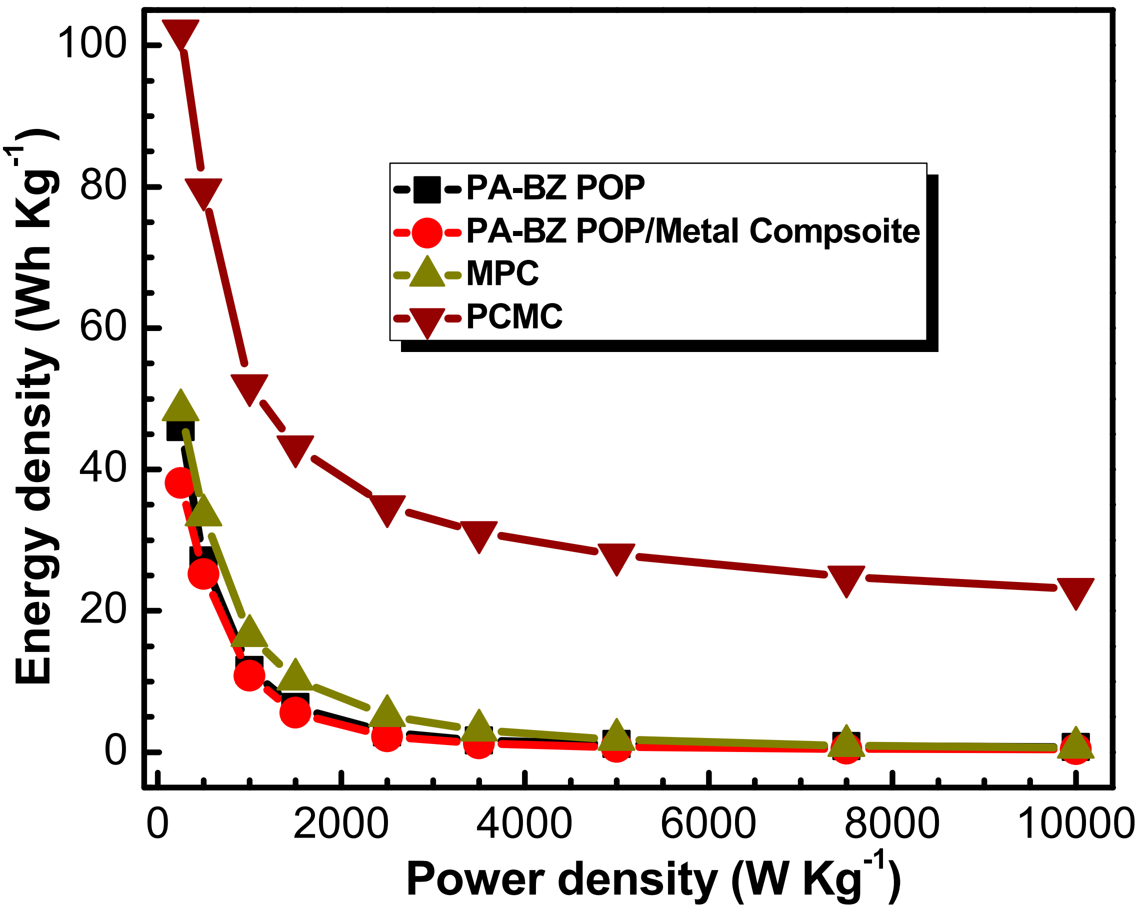

Figure S6: Ragone plot of P-AP-BZ POP, PA-BZ POP/Metal Composite, MPC, and PCMC.

Table S1: Comparison between the specific surface area/specific capacitance of our materials with those of previously reported materials for supercapacitor application.

Electrode	S_{BET} ($m^2 g^{-1}$)	Capacitance	Ref.
**PA-BZ POP**	**25**	**331 F g^{-1} at 0.5 A g^{-1}**	**This work**
**PA-BZ POP/Metal Composite**	**-**	**274 F g^{-1} at 0.5 A g^{-1}**	**This work**
**MPC**	**405**	**349 F g^{-1} at 0.5 A g^{-1}**	**This work**
**PCMC**	**1113**	**735 F g^{-1} at 0.5 A g^{-1}**	**This work**
Py-FFC-CMP	50	5.07 F g^{-1} at 0.5 A g^{-1}	S1
Py-FFC-CMP/CD-BZ	-	10.15 F g^{-1} at 0.5 A g^{-1}	S1
Py-FFC-CMP/poly(CD-BZ)	-	46 F g^{-1} at 0.5 A g^{-1}	S1
TPE-FFC-CMP	8	4.8 F g^{-1} at 0.5 A g^{-1}	S1
TPE-FFC-CMP/CD-BZ	-	7.53 F g^{-1} at 0.5 A g^{-1}	S1
TPE-FFC-CMP/poly(CD-BZ)	-	37.07 F g^{-1} at 0.5 A g^{-1}	S1
TBN-Py-CMP	473	31 F g^{-1} at 0.5 A g^{-1}	S2
TBN-TPE-CMP	1150	18.45 F g^{-1} at 0.5 A g^{-1}	S2
TBN-Car-CMP	762	18.90 F g^{-1} at 0.5 A g^{-1}	S2
TBN-Py-CMP/SWCNT	-	430 F g^{-1} at 0.5 A g^{-1}	S2
TBN-TPE-CMP/SWCNT	-	156 F g^{-1} at 0.5 A g^{-1}	S2
TBN-Car-CMP/SWCNT	-	53 F g^{-1} at 0.5 A g^{-1}	S2
POSS-A-POIP	426	152.5 F g^{-1} at 0.5 A g^{-1}	S3
POSS-F-POIP	452	36.5 F g^{-1} at 0.5 A g^{-1}	S3
Car-CTF-5-400	910.7	420 F g^{-1} at 50 mV s^{-1}	S4
Car-CTF-10-400	1248.6	388 F g^{-1} at 50 mV s^{-1}	S4
Car-CTF-5-500	1410.7	545 F g^{-1} at 50 mV s^{-1}	S4
Car-CTF-10-500	1334.4	470 F g^{-1} at 50 mV s^{-1}	S4
CoPc-CMP	-	17.7 F g^{-1} at 0.5 A g^{-1}	S5
CoPc-CMP	-	13.8 F g^{-1} at 1.0 A g^{-1}	S5
CoPc-CMP	-	11.2 F g^{-1} at 2.0 A g^{-1}	S5
Co-GPC	272.5	455 F g^{-1} at 0.5 A g^{-1}	S6
N-doped Cobalt@graphitized carbon material (ZC-600)	662.1	652 F g^{-1} at 1 A g^{-1}	S7
Carbon-ZSR	683	305 F g^{-1} at 1 A g^{-1}	S8

References

[S1] Samy, M.M.; Mohamed, M.G.; Mansoure, T.H.; Meng, T.S.; Khan, M.A.R.; Liaw, C.C.; Kuo, S.W. Solid state chemical transformations through ring-opening polymerization of ferrocene-based conjugated microporous polymers in host–guest complexes with benzoxazine-linked cyclodextrin. *J. Taiwan Inst. Chem. Eng*. **2021**. doi.org/10.1016/j.jtice.2021.10.010.

[S2] Samy, M.M.; Mohamed, M.G.; Kuo, S.W. Pyrene-functionalized tetraphenylethylene polybenzoxazine for dispersing single-walled carbon nanotubes and energy storage. Compos. Sci. Technol. **2020**, *199*, 108360. doi.org/10.1016/j.compscitech.2020.108360.

[S3] Mohamed, M.G.; Mansoure, T.H.; Takashi, Y.; Samy, M.M.; Chen, T.; Kuo, S.W. Ultrastable porous organic/inorganic polymers based on polyhedral oligomeric silsesquioxane (POSS) hybrids exhibiting high performance for thermal property and energy storage. *Microporous Mesoporous Mater.* **2021**, *328*, 111505. doi.org/10.1016/j.micromeso.2021.111505.

[S4] Mohamed, M.G.; EL-Mahdy, A.F.M.; Ahmed, M.M.M.; Kuo, S.W. Direct Synthesis of Microporous Bicarbazole-Based Covalent Triazine Frameworks for High-Performance Energy Storage and Carbon Dioxide Uptake. *ChemPlusChem* **2019**, *84*, 1767–1774. doi.org/10.1002/cplu.201900635.

[S5] Mei, L.; Cui, X.; Duan, Q.; Li, Y.; Lv, X.; Wang, H.G. Metal Phthalocyanine-Linked Conjugated Microporous Polymer Hybridized with Carbon Nanotubes as a High-Performance Flexible Electrode for Supercapacitors. *Int. J. Hydrogen Energy* **2020**, *45*, 22950-22958. doi.org/10.1016/j.ijhydene.2020.06.208.

[S6] Chen, Y.; Liu, F.; Qiu, F.; Lu, C.; Kang, J.; Zhao, D.; Han, S.; Zhuang, X. Cobalt-Doped Porous Carbon Nanosheets Derived from 2D Hypercrosslinked Polymer with CoN_4 for High Performance Electrochemical Capacitors. *Polymers* **2018**, *10*, 1339. doi.org/10.3390/polym10121339.

[S7] 75. Shao, D.; Wang, C.; Wang, L.; Guo, X.; Guo, J.; Zhang, S.; Lu, Y. A N-doped Cobalt@graphitized carbon material derived from ZIF-67 assisted polyvinylidene fluoride hollow fiber membrane for Supercapacitors. *J. Alloys Compd.* **2021**, *863*, 158682. DOI: 10.1016/j.jallcom.2021.158682.

[S8] Lei, Y.; Gan, M.; Ma, L.; Jin, M.; Zhang, X.; Fu, G.; Yang, P.; Yan, M. Synthesis of nitrogen-doped porous carbon from zeolitic imidazolate framework-67 and phenolic resin for high performance supercapacitors. *Ceram. Int.* **2017**, *43*, 6502–6510. DOI: 10.1016/j.ceramint.2017.02.072.
